# Comparison of four diagnostic techniques for *Cryptosporidium* detection in Qatar

**DOI:** 10.5339/qmj.2025.78

**Published:** 2025-08-20

**Authors:** Sallama Sabooni, Husam Salah, Rajvir Singh, Khloud Al-Qadi, Saad J. Taj-Aldeen, Emad Ibrahim

**Affiliations:** 1 Division of Microbiology, Department of Laboratory Medicine & Pathology, Hamad Medical Corporation, Doha, Qatar; 2 Heart hospital, Hamad Medical Corporation, Doha, Qatar; 3 Department of Biology, College of Science, University of Babylon, Iraq *Email: smustafa3@hamad.qa

**Keywords:** *Cryptosporidium*, stool microscopy, modified Kinyoun’s acid-fast stain, immunochromatography, polymerase chain reaction

## Abstract

**Background:**

*Cryptosporidium* is a common pathogenic parasite known to cause diarrhea in humans and animals, particularly in young children living in poor conditions. Although diarrheal disease is usually mild in immunocompetent individuals, it may progress into a life-threatening complication among the immunocompromised. Due to insensitive conventional diagnostic methods, the identification of *Cryptosporidium* can be inaccurate and challenging. The present study aimed to investigate the prevalence of cryptosporidiosis infection in Qatar by comparing four different diagnostic methods for detecting *Cryptosporidium* in human stool samples.

**Methods:**

From January 2018 to December 2019, stool samples from patients with various gastrointestinal symptoms were collected at the microbiology laboratory at Hamad General Hospital in Qatar for parasitic detection. The stool samples were tested using four diagnostic methods: routine microscopy, immunochromatography (ICT), multiplex polymerase chain reaction (PCR), and modified Kinyoun’s acid-fast stain (MKS).

**Results:**

In the evaluation of the four different detection methods, 36 (18%) out of 205 stool samples tested positive for *Cryptosporidium*, with detection rates of 18%, 15%, 7%, and 6% using PCR, ICT, MKS, and routine microscopy, respectively.

**Conclusion:**

The superior sensitivity of PCR and ICT supports their integration into routine diagnostics to improve the detection and public health surveillance of cryptosporidiosis in Qatar.

## 1. INTRODUCTION

*Cryptosporidium* species are common pathogenic protozoan parasites causing diarrheal diseases in humans and animals worldwide. Initially isolated in 1907, it was recognized as a global diarrheal pathogen in 1976.^1^ These organisms infect intestinal epithelial cells, leading to clinical manifestations ranging from self-limiting diarrhea to life-threatening illness in immunocompromised hosts. The intracellular protozoan coccidian parasite exhibits a life cycle that involves only one definitive host.^2,3^ Among the 44 known species, *C. hominis* and *C. parvum* dominate human infections, primarily via contaminated water or zoonotic contact.^4,5^ Although *C. parvum* is mainly a zoonotic pathogen, it can cause symptomatic cryptosporidiosis in humans.^6^ Furthermore, *C.meleagridis* (avian adapted) is the third most frequently identified *Cryptosporidium* species in humans. In addition, other pathogenic species such as *C. felis*, *C. muris*, *C. canis*, and *C. suis* have sporadically emerged in human cases of zoonotic outbreaks, especially following direct contact with infected animals.^2,7–9^

Globally, cryptosporidiosis remains a leading cause of childhood diarrheal mortality, particularly in developing nations with poor sanitation.^10–12^ The ASEAN (Association of Southeast Asian Nations) has classified cryptosporidiosis as the major intestinal protozoan infection among impoverished populations.^11^ Furthermore, it is listed as a category B pathogen by the CDC (Centers for Disease Control and Prevention)^10^ and the NIH (National Institute of Health) due to its potential to cause water contamination.^12^

Since the primary mode of transmission for this disease is through the fecal route, poor sanitation and low living standards are recognized as risk factors.^10,13^ Therefore, it has been recognized as the second leading cause of mortality associated with diarrhea among children under five years of age in sub-Saharan Africa and Southeast Asia.^7,14^ Although improved water management has led to reduced cases in industrialized countries, Qatar’s unique population structure and climate conditions continue to pose transmission risks. Additionally, frequent outbreaks have been linked to close animal contact, travel, and drinking or swimming in water contaminated with *Cryptosporidium* oocysts that are resistant to several disinfectants.^14,15^ In industrialized countries, improved water management practices have contributed to a reduction in cases of cryptosporidiosis among the general population.^10^ Recent studies conducted in Qatar have reported prevalence rates ranging from 0.05% in the general population to 15% among pediatric diarrheal cases, suggesting a significant degree of underdiagnosis when conventional methods are used.^16–18^

The pathogen primarily infects the mucosal epithelial cells of the gastrointestinal tract, leading to clinical manifestations that typically include abdominal cramps and diarrhea.^19^ In immunocompetent individuals, the disease manifests as asymptomatic to mild illness, characterized by transient and self-limiting diarrhea and abdominal symptoms. However, the disease can progress to a life-threatening condition with persistent symptoms among immunocompromised patients, leading to severe dehydration and wasting.^19^

Cryptosporidiosis manifests in three main forms: sporadic, chronic, and diarrhea with malnutrition in young children in developing countries.^10^ Sporadic infection is the most common form, often associated with water-related outbreaks, with self-limiting diarrhea in immunocompetent hosts. In contrast, chronic infection can lead to a life-threatening illness in immunocompromised patients, particularly those with HIV/AIDS infection.^10^ The US FDA (Food and Drug Administration) has approved nitazoxanide (Alinia^®^, manufactured by Romark Laboratories, Tampa, FL, USA) as an effective treatment for cryptosporidiosis.^20^ On the contrary, antiretroviral therapy has significantly reduced the mortality rates associated with *Cryptosporidium* infections in AIDS patients, particularly in medium-to high-income countries.^21^

Microscopic examination, although widely used,^22,23^ requires high oocyst concentrations (>50,000/mL) and exhibits poor sensitivity.^24^

Immunochromatographic tests^25^ show variable performance depending on parasite burden, while molecular methods remain underutilized in many settings despite their superior sensitivity.^26–29^

While some regional data are available from the Middle East and the GCC countries, reports remain scarce, with only a few studies reported from Jordan, Iraq, Lebanon, Israel, Egypt, Sudan,^30–35^ as well as selected GCC countries.^36,37^ In Qatar, cryptosporidiosis has been previously reported in three studies.^16–18^ Boughattas et al. investigated the frequency of intestinal parasites among pediatric patients with chronic diarrhea, using real-time polymerase chain reaction (PCR) mainly aimed at identifying the dominant species.^16^ A similar study using quantitative PCR assessed that the prevalence of *Cryptosporidium* species among food handlers and housemaids newly arrived in Qatar was 4.5%.^17^ Another epidemiological study estimated the prevalence of *Cryptosporidium* as 0.05% among 29,286 patient records referred for stool examination using conventional methods over 10-year period.^18^

Despite the existing regional^30–35^ and local reports, Qatar lacks data on comparative diagnostics for cryptosporidiosis. The aim of this study was to compare the diagnostic performance of four methods for detection of *Cryptosporidium* in human stool samples and to evaluate the need to integrate advanced methods alongside conventional techniques. Additionally, the study aims to investigate the prevalence of *Cryptosporidium* infections in Qatar among patients with diarrhea.

## 2. MATERIALS AND METHODS

### 2.1. Settings

Hamad Medical Corporation (HMC) serves as the main provider of secondary and tertiary healthcare in Qatar. HMC manages 12 hospitals, which include nine specialist hospitals and three community hospitals.

Samples from all HMC facilities, except Al Khor hospital, requested for ova and parasite examination are received at the Microbiology Laboratory of Hamad General Hospital (HGH).

### 2.2. Sample collection

A total of 205 stool samples (one sample per patient) were collected from both adult and pediatric inpatients and outpatients who presented with at least one clinical intestinal symptom such as diarrhea, abdominal pain, or loose stools, with a primary suspicion of infectious diarrheal disease. Samples were received at the Microbiology Laboratory of HGH for the examination of ova and parasites over a period of two years (January 2018 to December 2019). Stool samples were collected in sterile containers and transported to the microbiology laboratory within two hours of collection. In cases where testing was delayed, stool samples were stored at 4°C.^22^ The Institutional Board Review at HMC Medical Research Center waived the need for obtaining consent from patients.

### 2.3. Inclusion and exclusion criteria

Soft, loose, and diarrheal stool samples were obtained from both inpatients and outpatients. These samples were selected to ensure the representation of individuals experiencing gastrointestinal symptoms associated with potential *Cryptosporidium* infection. Stool samples with formed and semi-formed consistencies, as well as those obtained from other facilities, were excluded.

### 2.4. Direct microscopy

Approximately 1–2 mg of stool was mixed on a clean glass slide with 1–2 drops each of normal saline (0.85% NaCl) and D᾽Antoine’s iodine stain (Canada Wide Scientific Ltd, Ottawa, Canada), prepared according to the manufacturer’s protocol. A coverslip was then placed on the slide and examined under 20× and 40× objective lenses of a light microscope for the presence of *Cryptosporidium* oocysts^22^

### 2.5. Formalin-ether acetate (FEA) concentration technique

Soft and loose stool samples, weighing more than two grams, were concentrated using the FEA technique. To 15 mL of 10% formalin, five milliliters of ethyl acetate (VWR International SAS, France) were added to the stool samples and mixed thoroughly. The mixture was then centrifuged at 3,000 RPM for 10 minutes, and the supernatant was discarded. Two drops of the resulting sediment were smeared directly onto a clean glass slide, followed by the addition of 1–2 drops of D’Antoine’s iodine stain. The preparation was covered with a coverslip, and the slide was examined under a light microscope using 20× and 40× objective lenses to detect *Cryptosporidium* oocysts. For stool samples weighing less than two grams, such as those obtained from infants, the direct microscopy method was used.^22^

### 2.6. Modified Kinyoun’s acid-fast stain (MKS)

Stool samples were smeared onto a clean glass slide and fixed on a hot plate at 55°C for 10 minutes, followed by staining with Kinyoun’s carbol fuchsin for one minute. The smears were immersed in 50% ethanol for one minute and rinsed with clean tap water. The smears were then decolorized with 1% hydrochloric acid for two minutes and rinsed with water. Finally, the smears were counter-stained with methylene blue for 15–20 minutes, according to their thickness, and rinsed with water. The slides were blot dried with bibulous paper and examined under a light microscope with a 100× (oil immersion) objective lens for the presence of *Cryptosporidium* oocysts.^38^

### 2.7. Immunochromatography (ICT) test

The Crypto + Giardia rapid ICT assay (Biotech, Spain) was used for the detection of *Cryptosporidium* parvum following the manufacturer’s protocol. Unpreserved stool samples were initially stored at 2–8°C for 1–3 days and frozen at −20°C for longer storage. Approximately 0.5 g of stool (or 125 μL for liquid stool) was diluted in a dilution buffer and mixed thoroughly. Three drops of suspension were added to the test window of the ICT device, and the results were observed after 10 minutes. The appearance of a visible green control line (C) on the device indicated that the test was successfully performed. A positive reaction appeared as a visible red band in the test window (T), regardless of its intensity, indicating the presence of Giardia/*Cryptosporidium*. The test was interpreted as negative when no reaction was observed in the test window, while a green control line (C) was visible after 10 minutes. According to the manufacturer’s specifications, the sensitivity of the kit is 94.3%, with a specificity of 100%, a positive predictive value (PPV) of 100%, and a negative predictive value (NPV) of 97.8%. There was no cross-reactivity with other gastrointestinal parasites that may occasionally be present in feces. The kits were stored at room temperature according to the manufacturer’s instructions.

### 2.8. Molecular testing (PCR)

Molecular testing was performed using Allplex™ GI-Parasite Assay (Seegene, Seoul, Korea). This assay was used for the detection and identification of Blastocystis hominis, *Cryptosporidium* spp. (*C.meleagridis*, *C. hominis*, and *C. parvum*), Cyclospora cayetanesis, Dientamoeba fragilis, Entamoeba histolytia, and Giardia lambila, based on the amplification and sequencing of the 18S rRNA. Stool samples were stored at −20°C until use. They were thawed at room temperature and pretreated with the QIA GEN mini kit (Qiagen, USA), where approximately 0.2 g of stool sample was eluted in 100 μL of elution buffer to remove contaminants and PCR inhibitors, as a pre-analytical step performed according to the manufacturer’s instructions. DNA extraction was performed using the STARMag 48 × 8 Tissue cartridge kit (Seegen, Seoul, Korea), with the Microlab NIMBUS IVD automated nucleic acid extraction instrument (Hamilton, USA). Real-time PCR was performed using the CFX96™ thermocycler (Bio-Rad, USA), according to the manufacturer’s instructions, and the results were then analyzed using Seegene viewer software (Seegene, Korea). The PCR kit included internal, positive, and negative controls to confirm the validity of the results. The cycle threshold (Ct) values were used to interpret the results, with average Ct values generated automatically. Ct values of ≤43 indicated a positive result, while values >43 were considered negative. Previous studies have reported that sensitivities for the Allplex™ GI-Parasite Assay kit range from 91.5% to 99.0%, with specificities from 98.3% to 100%. According to the manufacturer’s manual, the Allplex™ GI-Parasite kit does not exhibit cross-reactivity with other intestinal pathogens.^28,39–42^

### 2.9. Statistical analysis

Test results were analyzed using SPSS software (SPSS 22 Inc., Chicago, USA). Frequencies and percentages were calculated for categorical variables. The association between *Cryptosporidium* positivity and demographic factors (age group, gender, nationality, season) were analyzed using chi-square tests. The probability (p) value of ≤0.05 (two tailed) was considered as statistically significant. The diagnostic sensitivity, specificity, PPV, and NPV of various diagnostic assays were calculated by using the PCR results as the reference standard. The sensitivities and specificities of various diagnostic techniques were measured based on the results of the Allplex™ GI-Parasite Assay.

## 3. RESULTS

### 3.1. Patients’ characteristics

The study included a total of 205 patients, with 113 males (55%) and 92 females (45%). The patients’ ages ranged from 5 months to 71 years (mean 33.6 years). The patient population residing in Qatar was diverse: Qatari nationals (*n* = 88, 43%), the Middle East (*n* = 56, 27.3%), Southeast Asia (*n* = 50, 24.3%), Africa (*n* = 6, 3%), and Europe (*n* = 5, 2.5%).

### 3.2. Diagnostic performance of methods

Among the 205 stool samples analyzed, 36 (18%) were positive for *Cryptosporidium*. The detection rates for the different methods were as follows: 18% for PCR, 15% for ICT, 7% for MKS, and 6% for routine microscopy. The diagnostic performance of the four methods used for detecting *Cryptosporidium* infection is summarized in Table 1.

### 3.3. Direct microscopy

Of the 205 specimens screened by microscopy, *Cryptosporidium* oocysts were detected in 12 (6%) samples using wet mount preparations stained with iodine. This method had a sensitivity of 33% and a specificity of 100%. Furthermore, these samples were tested positive using MKS, ICT, and PCR methods. Among the 193 negative samples, 3 (1.5%) were found to be positive using MKS, 19 (10%) were positive using ICT, and 24 (12%) showed positive results using PCR.

### 3.4. MKS

*Cryptosporidium* oocysts were detected in 15 (7.3%) out of the 205 smears stained with MKS, with a sensitivity of 41.7% and a specificity of 100%. These samples also tested positive using ICT and PCR. Among the 190 negative samples, 16 were positive by ICT and 21 were positive by PCR. Additionally, three positive samples by MKS showed negative results when assessed by direct microscopy.

### 3.5. ICT

In the specimens screened by ICT, *Cryptosporidium* antigen was detected in 31 out of 205 (15%) specimens, with a sensitivity of 86.1% and a specificity of 100% (Table 1). Among the positive ICT samples, 16 specimens yielded negative results with MKS, while 19 were not detected through direct microscopy. Out of the 174 negative samples, five (3%) were also found to be positive using PCR.

### 3.6. PCR

PCR analysis detected *Cryptosporidium* DNA in 36 (17.5%) out of 205 samples. Of these, 25 samples were negative when assessed by direct microscopy, 22 were negative by MKS, and 5 were negative by ICT; however, all were confirmed positive by PCR. The five samples that were negative by ICT but positive by PCR were confirmed using another molecular technique, Biofire (BioFire Diagnostic, LLC, Utah, USA). The median PCR cycle threshold (Ct) value was 33.89, ranging from 26.95 to 40.24.

### 3.7. Occurrence of *Cryptosporidium* infection

From the 36 patients who tested positive for *Cryptosporidium*, 14 (39%) were under five years of age, six (17%) were between 5 and 10 years, seven (19%) were between 10 and 20 years, six (17%) were between 20 and 40 years, and three (8%) were >40 years (Table 2). Statistical analysis revealed a significant association between age groups and the incidence of *Cryptosporidium* infection (*p* = 0.04).

Among the positive cases, 22 (61.1%) were male patients, while 14 (38.9%) were female patients (Table 2). However, the difference in infection rates by gender was not statistically significant (*p* = 0.37). Of the positive samples, 22 (61.1%) belonged to Qatari patients and 14 (38.9%) were detected in patients of other nationalities. The evaluation of the regression model showed a statistically significant difference between these groups (*p* = 0.02).

**Figure 1:**
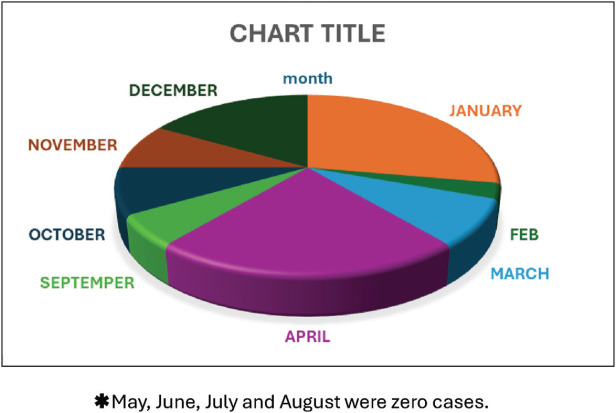
Prevalence of cryptosporidiosis during the year 2019 (chi-square = 41/3, df = 11, *p* = 0.001).

The present study also showed that *Cryptosporidium* infections followed a seasonal pattern, with higher infection rates observed during the winter and rainy seasons (October to April) and lower rates observed during the summer months (June to September) (Figure 1), indicating a significant difference in the distribution of the infection across months (*p* = 0.01).

## 4. DISCUSSION

This study investigated the prevalence of *Cryptosporidium* infections in Qatar and compared the diagnostic performance of four different techniques for detecting *Cryptosporidium* in human stool samples. Our findings revealed a higher prevalence than previously reported studies conducted in Qatar. Boughattas et al. investigated the prevalence of *Cryptosporidium* among symptomatic pediatric patients with chronic diarrhea and reported an incidence rate of 15.1%.^16^ Additionally, another study reported that stool screening of food handlers and domestic workers aged 18–56 years showed *Cryptosporidium* in 4.5% of cases.^17^ These results should be examined with caution, since, unlike our cohort, these individuals were apparently healthy and asymptomatic.

The incidence of *Cryptosporidium* infection was higher in males (61%), as reported in previous studies.^18,43^ A study conducted in Saudi Arabia by Hawash et al. linked the higher incidence of cryptosporidiosis in males to their direct contact with farm animals.^43^ In contrast, other studies have reported higher rates of *Cryptosporidium* infections in females who also had direct contact with animals.^44,45^ These findings may be influenced by local factors and require further investigation.

Additionally, the present study revealed that children under five years of age have twice the infection rate compared to older children (5–10 and 10–20 years). This observation may be attributed to several factors. Specifically, young children under five years of age have less mature immune systems compared to older children, making them more susceptible to infections. Additionally, their behaviors, such as frequent hand-to-mouth contact and poor adherence to hygiene practices, increase their exposure to pathogens. This finding is in agreement with studies conducted in Qatar and elsewhere.^16,36,46–48^ Interestingly, the incidence of cryptosporidiosis among Qatari nationals (61%) was higher than that among other expatriates (39%), which was not expected, although this has been previously reported in a local epidemiological survey.^16^

Examining the seasonality of *Cryptosporidium* detection in relation to the reporting months, we found that the prevalence was higher during the winter and rainy seasons compared to other periods. This trend was similarly observed in previous studies from Qatar,^16^ Kuwait,^49^ Saudi Arabia,^45^ and sub-Saharan Africa.^10^ These regions share similar climatic conditions, suggesting that environmental factors, such as increased rainfall and lower temperatures, may contribute to the increased prevalence observed during the winter and rainy seasons. However, our findings contradict another study from Saudi Arabia, which found the highest rates of *Cryptosporidium* infection occurring during the fall and spring seasons.^44^ Another study from Jordan showed that the highest prevalence of *Cryptosporidium* was reported during the warmer months, from May to September.^50^

The most common risk factors associated with *Cryptosporidium* infection globally are direct contact with domestic animals and environmental factors, such as unsafe water sources, malnutrition, and immunosuppression.^19,51,52^ The observed seasonal prevalence during lower temperatures may be attributed to increased outdoor activities and contact with animals during these months compared to the hotter summer period. This observation is supported by a previous study from Kuwait, which reported a high prevalence of *Cryptosporidium* linked to the consumption of contaminated water in winter desert camps, where a large number of water storage tanks were used.^49^ In Qatar, no seasonal studies have been conducted to determine the relationship between meteorological and other factors, such as water contamination and animal contact, in relation to the transmission of *Cryptosporidium*.

Additional risk factors identified in previous prevalence reports of cryptosporidiosis in the GCC region found that the most vulnerable groups were children under five years of age and the immunocompromised.^36^ Furthermore, Ahmed et al. showed that expatriate workers were the source of imported *Cryptosporidium* infection via food handling and poor hygiene.^36^ However, expatriates from regions with a high rate of parasitic infections, such as Southeast Asia and North and sub-Saharan Africa, may be a possible source of *Cryptosporidium* transmission in Qatar.^16,18^

Regarding the accuracy of diagnostic tests, the PCR method emerged as the most sensitive test compared to the other three diagnostic techniques used in this study. The PCR method detected the highest positive samples, with 36 out of 205 (17.5%), compared to direct microscopy, MKS, and ICT (6%, 7.3%, and 15%, respectively). These results are in accordance with previous studies.^23,32,37,51,52^ On the contrary, direct microscopy and MKS showed the lowest sensitivity rates among the four methods tested, with sensitivity rates of 33% and 41.7%, respectively. Despite their low sensitivity, both methods showed 100% specificity (Table 1). It is important to highlight that the sensitivity of both techniques can be improved by testing multiple samples repeatedly. Previous reports have shown that the probability of detecting parasites in a single stool specimen may be as low as 50–60%, whereas it can reach to >95% if three stool samples are tested.^53,54^

Additionally, the ICT method proved to be more beneficial in our study than direct microscopy and MKS, showing a sensitivity of 86%. The improved sensitivity of the ICT may be due to its ability to detect lower levels of *Cryptosporidium* oocysts, which can be challenging with direct microscopy. This may occur possibly after an anti-parasitic treatment.^24^ It is worth noting that the kit used in our study specifically targets C. parvum, the predominant species in Qatar,^16^ which likely contributed to the high sensitivity observed with the ICT method in our study. Additionally, these results cannot be extrapolated elsewhere since pathogen epidemiology may differ. These findings are in agreement with those from previous studies.^43,55,56^ However, previous studies have reported lower sensitivities and specificities of ICT when using both similar and different kits.^24,57^

Currently, a wide range of commercial multiplex PCR assays have been developed to overcome the limitations of conventional microscopic and staining techniques used for parasite detection in stool samples.^29,58^ These PCR assays offer several advantages over traditional methods. Firstly, they provide increased sensitivity and specificity, particularly in populations with a low rate of parasitic infections. The Allplex™ GI-Parasite Assay showed a high sensitivity of 96.5% and a specificity of 98.3%.^28^ Secondly, the possibility of multiplex PCR assays allows the detection of various organisms and species using multiplex gene targets. Additionally, these assays enable the detection of bacterial and viral co-infections within the same test panel, providing a comprehensive diagnostic solution for patients with mixed infections.^28^

However, PCR assays may have some limitations in detecting some protozoan parasites (e.g., Cystoisospora belli) in stool samples.^40^ Therefore, when selecting the most appropriate assay, it is crucial to consider several factors, including laboratory workflow, test performance, and the specific patient population being tested.^41^ Since standard methods for diagnosing *Cryptosporidium* lack sensitivity, we recommend the inclusion of PCR and ICT techniques alongside conventional methods, given the proven insensitivity of conventional methods (microscopy: 6% detection; MKS: 7% detection).

## 5. CONCLUSION

In summary, this study highlighted the superiority of PCR assays in terms of sensitivity and specificity for the detection of *Cryptosporidium* compared to ICT, MKS, and direct microscopy. Additionally, the study showed that infection rates of *Cryptosporidium* were higher in children under five years of age compared to other groups. Moreover, a significantly higher incidence of infections was observed during the winter and rainy seasons, which warrants further investigation.

Considering the increased prevalence of cryptosporidiosis in Qatar, improved detection methods for *Cryptosporidium* are warranted to ensure accurate diagnosis and prevention of the disease. Therefore, the introduction of ICT and PCR assays for detecting *Cryptosporidium* is recommended, as these methods are less time-consuming, simple to perform, and require minimal training. Future research using genomic sequence typing will be useful to understand the molecular epidemiology and species diversity of *Cryptosporidium* in Qatar. Additionally, more efforts should focus on improving the accessibility of molecular diagnostics in resource-limited settings. We also recommend the implementation of targeted interventions to reduce the transmission of cryptosporidiosis, including periodic molecular screening of high-risk food products, enhanced surveillance of food handlers during seasonal outbreaks, and routine testing of severely immunocompromised patients (e.g., hematology/oncology and transplant recipients) given their risk of severe chronic infections.

## AUTHORS’ CONTRIBUTIONS

SS was responsible for the study design, implementation of technical works, interpretation, and analysis of the results, and wrote the manuscript. HS assisted in designing, revision, editing, and approved the submitted version. RS performed the statistical analysis. KA provided technical assistance. EI reviewed the methodology and results. ST reviewed and edited the final manuscript. All authors read and approved the final version of the manuscript.

## ACKNOWLEDGMENT

We thank Dr Hamad Abdelhadi, Ms Fatma Al-Mohannadi, and Mr Abdullah Aldaw for their support during the study period.

## FUNDING SOURCES

This study was supported by the Medical Research Centre at Hamad Medical Corporation (grant MRC/172510/17 to Sallama Sabooni).

## COMPETING INTERESTS

None of the authors have any conflicts of interest to disclose.

## ETHICS APPROVAL

This study was approved by Hamad Medical Corporation Research Center and the IRB Ethical Committee (reference number MRC/172510/17). All methods were performed in accordance with the guidelines and regulations outlined in the SOP (Standard Operating Procedure) for Microbiology Laboratory at Hamad Medical Corporation.

## Figures and Tables

**Table 1.** Sensitivity and specificity rates of various diagnostic tests with respect to the PCR test.

**Method**	**Positive (n, %)**	**Negative (n, %)**	**Sensitivity (95% CI)**	**Specificity (95% CI)**	**PPV (%)**	**NPV (%)**
Microscopy	12/205 (5.9%)	193/205 (94.1%)	33% (18.5–50.9%)	100% (97.8–100%)	100%	87.5%
MKS	15/205 (7.3%)	190/205 (92.7%)	41.7% (25.5–59.2%)	100% (97.8–100%)	100%	88.9%
ICT	31/205 (15.1%)	174/205 (84.9%)	86.1% (70.5–95.3%)	100% (97.8–100%)	100%	97.1%

PPV: Positive predictive value, NPV: negative predictive value, MKS: modified Kinyoun’s acid-fast stain, ICT: immunochromatography.

**Table 2.** Prevalence of *Cryptosporidium* based on gender and age groups.

		**No. of positive samples (%)**	**No. of negative samples (%)**	**Total no. of samples (%)**
Gender^*^	Female	14 (38.9)	78 (46.2)	92 (44.9)
	Male	22 (61.1)	91 (53.8)	113 (55.1)
Age (years)^#^	<5	14 (38.9)	36 (21.3)	50 (24.4)
	5–10	6 (16.7)	22 (13.0)	28 (13.7)
	10–20	7 (19.4)	22 (13.0)	29 (14.1)
	21–40	6 (16.7)	50 (29.6)	56 (27.3)
	>40	3 (8.3)	39 (23.1)	42 (20.5)

^*^Chi-square = 0.63, df = 1, = 0.42.

^#^Chi-square = 9.9, df = 1, *p* = 0.04.
